# How Does the COVID-19 Pandemic Affect People’s Willingness to Pay for Health in the Short and Long Term? A Longitudinal Study during and after the COVID-19 Pandemic in China

**DOI:** 10.3390/ijerph19031568

**Published:** 2022-01-29

**Authors:** Wei Song, Taiyang Zhao, Ershuai Huang

**Affiliations:** 1 Yatai School of Business Management, Jilin University of Finance and Economics, Changchun 130117, China; 114074@jlufe.edu.cn; 2 School of Philosophy and Sociology, Jilin University, Changchun 130012, China; 3 School of Business Administration, Henan Polytechnic University, Jiaozuo 454003, China; huanges18@mails.jlu.edu.cn

**Keywords:** COVID-19 pandemic, willingness to pay for health, fear of death, self-esteem

## Abstract

The COVID-19 pandemic has posed a substantial threat to people’s lives and aroused health concerns. This study aims at exploring the following questions. First, how does the COVID-19 pandemic affect people’s willingness to pay for health (WPH) in the short and long term? Second, what is the psychological mechanism underlying such an effect? Finally, what are the boundary conditions for this effect? To answer these questions, we conducted three longitudinal surveys. The first survey was launched in February 2020—the time of the most serious outbreak of COVID-19 in China. Data were obtained from 1548 participants through questionnaires on an online survey platform. The sample covered 297 prefecture-level cities in 31 provincial administrative regions. Subsequently, we conducted two follow-up surveys in August 2020 and July 2021. The samples of these surveys were randomly selected from the sample of the first survey. The findings showed that the pandemic promoted people’s WPH in the outbreak period. The fear of death and self-esteem mediated and moderated this effect, respectively. Moreover, the effect persisted for six months after the COVID-19 pandemic had been brought under control (August 2020). However, the effect disappeared after a year and a half (July 2021). These results indicate that the COVID-19 pandemic promoted people’s WPH and that this effect was sustained in the short term after the pandemic had been brought under control but not in the long term.

## 1. Introduction

At the time of writing, the COVID-19 pandemic has been ravaging the world for two years. In those years, how the pandemic has affected the health behaviors of individuals has become a controversial issue. On one hand, some studies have found that residents took some unhealthy behaviors to cope with the negative emotions caused by the pandemic, including alcohol consumption [1,2,3], overeating [4,5], and tobacco use [6,7,8]. On the other hand, other studies have found that the pandemic improved residents’ health awareness [9,10] and self-protective behaviors [11,12]. Here, we argue that such confounding results may to some extent be due to the fact that existing studies primarily focused on the outbreak period of the pandemic [13,14] rather than adopting a longitudinal perspective. Specifically, during the outbreak period, the aforementioned healthy and unhealthy behaviors might simply constitute situational responses to the threat caused by the pandemic [2,11]. However, the long-term impact of the pandemic on residents’ health behaviors has yet to be fully understood. In particular, whether such effects will persist once the pandemic is under control has not yet been examined. Therefore, conducting longitudinal research to explore the short- and long-term impact of the pandemic on residents’ health behaviors is essential to further develop the literature in related fields.

In the present study, we choose residents’ willingness to pay for health (WPH) as our dependent variable in light of the following considerations. First, the consumption of goods that are beneficial to health is an important means of securing health. Compared to the temporary self-protection behaviors that have been investigated by previous research [11,12], WPH can reflect residents’ more general health awareness in their daily life. Second, WPH has been found to be more related to economic recovery after the pandemic. During the past 2 years, people have been gradually paying more attention to saving rather than consumption to cope with the economic downturn caused by the pandemic [15,16]. If the pandemic could lead to an increase in residents’ WPH, the health industry will usher in an important developmental opportunity and help the economy recover. Finally, studying the impact of the pandemic on WPH is consistent with the value orientation of promoting residents’ public health. In recent years, governments and scholars across the world have been paying increasing attention to the promotion of healthy behaviors for the public. Most of the existing literature has primarily focused on the negative impacts of the pandemic, such as anxiety, depression, and posttraumatic stress disorder (PTSD) [17,18]; however, this study focuses on the positive effects of the pandemic and has a more constructive value for public health policy. Given the aforementioned facts, can the COVID-19 pandemic serve as an opportunity to improve people’s health-related consumption behaviors? If so, how long will such a positive effect last? These are matters worth exploring. Against this background, the present study will explore the following questions. First, how does the COVID-19 pandemic affect people’s WPH in the short and long term? Second, what is the psychological mechanism underlying such an effect? Finally, what are the boundary conditions for this effect? To answer these questions, we constructed a theoretical model in which the severity of the pandemic (SP) acts as the independent variable, WPH as the dependent variable, the fear of death (FD) as the mediating variable, and self-esteem as the moderating variable.

This study employs a longitudinal design, adopting the method of a follow-up investigation. Specifically, we first collected data during the outbreak period to analyze the situational impact of the pandemic on people’s WPH. Subsequently, we conducted two follow-up surveys, after the pandemic had been brought under control, to analyze its short- and long-term impact. The short-term impact refers to the impact in a relatively short period just after the pandemic had been brought under control; the long-term impact refers to the impact at a more recent time after the pandemic had been assessed to have been controlled for a relatively long time. Thus, our first follow-up survey was conducted within 6 months after the first survey during the outbreak period, and the second follow-up survey was conducted 18 months after the first survey.

Furthermore, we assert that China serves as an ideal context for a longitudinal study of the impact of COVID-19 to address such issues. First, China has undergone all the possible stages: from the beginning of the outbreak to the pandemic being brought under control. This characteristic is useful for longitudinal studies that seek to compare the impact of pandemics on people’s WPH over different periods. Second, the pandemic in China has been under control for a long time, which helps us examine the duration of the effect of a pandemic on WPH. Finally, there are significant regional differences in the outbreak in China, with some areas having more cases (e.g., Wuhan) and others having virtually no cases (e.g., Tibet). This makes it easier to analyze the impact of the pandemic by comparing regional differences.

## 2. Theoretical Background and Hypotheses

### 2.1. The Promoting Effect of Pandemic on Residents’ WPH

The WPH reflects people’s intention to maintain or achieve health by consuming health-related products. Previous studies have found that environmental risks that threaten people’s health play a significant role in promoting their health behaviors [19,20]. As a public health emergency, the most significant impact of COVID-19 on people is the threat to their lives and health. Studies on consumer behavior have demonstrated that when an aspect of a consumer’s self is threatened, the consumer is more inclined to buy products or services that can help them compensate for it in what is called “compensatory consumption behavior” [21]. For example, when an individual feels a threat to their physical attractiveness, they will increase their consumption of fashion, cosmetics, and bodybuilding to improve their appearance [22]. Similarly, when an individual suffers a state of powerlessness, they will show more willingness to buy status goods [23]. During the COVID-19 outbreak, the numbers of confirmed cases and deaths were constantly surging, a treatment plan had not yet been formed, and effective drugs remained unproven, thus threatening people’s lives and health. According to the concept of compensatory consumption, in such a situation, individuals are more likely to increase their health consumption expenditure to deal with the threat to their health. In China, there are regional differences in the outbreak of COVID-19 (i.e., SP varies in different regions); thus, people in different regions would likely feel different levels of threat from the pandemic. Consequently, their related WPH would vary. Based on this deduction, we propose Hypothesis 1.

**Hypothesis** **1** **(H1).** *SP will be positively correlated with people’s WPH; that is, the more serious the pandemic is, the stronger people’s WPH will be.*

### 2.2. Terror Management Theory and the Mediating Effect of FD

Terror management theory (TMT) is a theory that explains how people cope with FD when facing death-related information [24]. Empirical studies based on TMT have found that FD increases people’s consumption of products that can improve their health [25]. This is because, according to TMT, high self-esteem can effectively act as a buffer against anxiety aroused by death, and a healthy body is a source of self-esteem [26]. In the context of COVID-19, the more severe the pandemic is, the higher the risk of infection for the people is, and the more they are exposed to scenes and information related to severe illness and death. Therefore, SP can increase people’s FD. To deal with this fear, individuals take various measures to strengthen their coping resources. As the ultimate resource for coping with disease and death is health, people evince more WPH to reduce their FD. Based on this logic, we propose the following hypothesis.

**Hypothesis** **2** **(H2).** *FD will play a mediating role in the relationship between SP and people’s WPH. Specifically, the more serious the pandemic is, the stronger people’s FD will be, and the stronger their WPH will be.*

### 2.3. The Moderating Role of Self-Esteem

Self-esteem describes the self-cognition of whether people’s evaluations of and attitudes toward themselves are positive or negative. Since everyone pursues a positive evaluation of themselves, the pursuit of high self-esteem is a basic motivation of individuals [27]. Previous studies have found that a higher level of self-esteem can help people offset the FD that is aroused by mortality salience [28]. Self-esteem can defend against FD because individuals with high self-esteem have more psychological resources and can resist the negative effects of FD more effectively. Based on such an effect on self-esteem, previous studies have found that individuals who suffer from mortality salience will engage in some behaviors to enhance self-esteem. For example, Rudert et al. found that individuals are less likely to experience regret after mortality salience because regret will lower their self-esteem [29]. Goldenberg et al. found that women were more inclined to maintain their body shape and high physical self-esteem in situations involving mortality salience [26]. Therefore, self-esteem comprises an important resource for individuals to cope with FD. As we demonstrated in the earlier section, improving health can be viewed as a way to reduce FD by enhancing physical self-esteem. When facing FD, individuals who have low self-esteem are more likely to feel that they have fewer resources to deal with FD compared to those who have high self-esteem. As a consequence, individuals with low self-esteem are more likely to search for effective methods to improve their self-esteem and, thus, show a higher WPH. Therefore, the following hypothesis is proposed.

**Hypothesis** **3** **(H3).** *Self-esteem will play a negative moderating role in the relation between FD and WPH. Specifically, compared to individuals with high self-esteem, individuals with low self-esteem will have a higher WPH when facing the FD caused by the pandemic.*

### 2.4. Short or Long Term?

In the previous sections, we showed how the pandemic awakened people’s FD and, consequently, increased their WPH. However, would this effect persist in the short and long term after the pandemic has been brought under control? We posit that this effect will continue in the short term once the pandemic is under control because its impact on people’s psychology will still persist for a time. Studies on PTSD have found that psychological impact can last for some time after the end of a period of stressful events [30,31]. Specifically, even if the pandemic is under control, residents in areas with a higher level of SP will still feel its impact more than those in areas with a lower level of SP and will retain a stronger FD, thus maintaining a higher WPH. However, FD is an emotion that changes with the circumstances. Over time, people’s emotions will eventually fade, and stressful trauma will be slowly alleviated [32]. Therefore, after the pandemic has been brought under control for a relatively long time, people’s FD will gradually dissipate, their way of life will gradually return to that before the pandemic hit, and their WPH will gradually decline. In summary, the impact of SP on people’s WPH will eventually disappear once the pandemic has been under control for a long period. Based on this logic, the following hypotheses are proposed.

**Hypothesis** **4a** **(H4a).** *In the outbreak period, SP will have a significant impact on people’s WPH.*

**Hypothesis** **4b** **(H4b).** *In the short-term period after the pandemic has been brought under control, the impact of SP on people’s WPH will still be significant.*

**Hypothesis** **4c** **(H4c).** *In the long-term period after the pandemic has been brought under control, the impact of SP on people’s WPH will disappear.*

**Figure 1:**
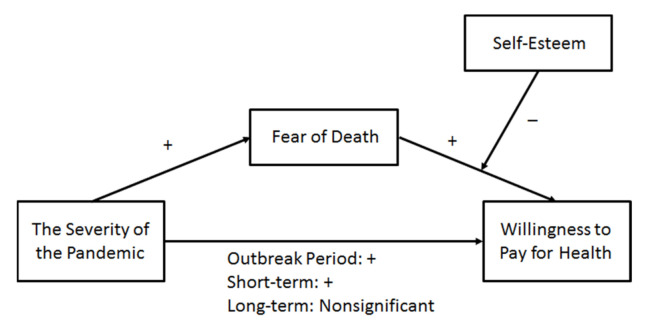
Theoretical model. Note: + means a significant positive effect, —means a significant negative effect.

Based on this discussion, Figure 1 presents the proposed conceptual model of this study.

## 3. Methods

### 3.1. Survey Design and Participants

This study adopted a longitudinal survey design and conducted three online questionnaire surveys at different stages of the COVID-19 pandemic in China. The infection first broke out in China in January 2020 and reached its peak on 12 February 2020 (15,152 local cases were confirmed that day). Subsequently, the rate of infection gradually decreased and was brought under control by 18 March 2020 (eight new cases were confirmed that day). In the following year and a half, although the rate of infection occasionally showed a sporadic trend, it remained within a good margin [33].

Our first survey was conducted during the most serious outbreak period (February 2020) on an online survey platform named Credamo. Users voluntarily registered an account on this platform to complete questionnaires. The questionnaire was randomly distributed over the app that was used by registered users, who earned a monetary reward by completing the questionnaire to show appreciation for the respondents’ participation. In this study, 1550 users were randomly selected from 31 provinces in China (50 users in each province). In the questionnaire, we designed two detection items to test whether the participants answered the questions carefully. In these detection items, we asked participants to address the options that we specified. If participants answered incorrectly, it meant that they had not answered carefully and were removed from the sample. After excluding two such respondents, we obtained a total of 1548 samples, covering 297 prefecture-level cities across 31 provincial administrative regions in China. The sample included 685 female (44.3%) and 863 male (55.7%) participants. Their ages were between 18 and 79 years old; their income ranged from 1000 to 20,000 RMB per month. Most of the respondents had completed a college- or university-level program (76.6%).

Six months later, in August 2020, we launched our first follow-up survey when the COVID-19 pandemic in China had entered a stable period. The questionnaires were randomly distributed to 500 of the participants in the first survey. Of these, 463 questionnaires were filled out and submitted, giving a completion rate of 92.6%. These data were used to analyze the short-term impact of the pandemic. One year later, we conducted the second follow-up survey in July 2021 using the same procedure as that for the first follow-up survey. In total, 500 questionnaires were distributed, and 427 were recovered for a completion rate of 85.4%. These data were used to analyze the long-term impact of the pandemic.

### 3.2. Measure

Because SP varied across regions, we used data on the pandemic in the region where the participants lived as an indicator of SP. We selected the following indicators published by the National Health Commission of the People’s Republic of China [33]: the cumulative number of confirmed cases per city (CCC), the number of new confirmed cases per city (NCC), the cumulative number of suspected cases per city (CSC), and the number of new suspected cases per city (NSC). Subsequently, we converted these four indicators into standard scores and took the average as the independent variable. As the numbers of deaths caused by COVID-19 were zero in most cities, we did not use this indicator. The independent variable herein was WPH, and we used the following items as its measures: (1) I will take the initiative to buy goods that are beneficial to my health; (2) when making consumption decisions, I will consider health factors to a great extent; and (3) I would rather pay higher prices to buy healthier goods. The mediating variable was FD, which was measured using the following items: (1) I feel that death is not far from my life; (2) I feel fear when I think of death; and (3) some things in my life have reminded me of death recently. For the measurement of self-esteem, which served as the moderating variable, we used the 10-item scale developed by Rosenberg (see Table A1 in Appendix A) [27].

In addition, we collected the following indicators of the participants and used them control variables for our analysis: gender, age, average monthly personal income (AMPI), average monthly household income (AMHI), average monthly personal expenses (AMPE), average monthly household expenditure (AMHE), and educational level.

We used the SPSS 24.0 and Amos 24.0 software programs, developed by IBM Corp, Armonk, NY, USA to analyze the reliability and validity of the four variables. Table 1 shows the results. The result of a confirmatory factor analysis indicated that all the standardized factor loadings for each item in the four variables were higher than 0.615. The values of Cronbach’s α and the composite reliability (CR) of the four variables were higher than 0.700; all the AVE values were higher than 0.500. These data suggest that the measurements used in this study had the required reliability and validity.

## 4. Results

### 4.1. Main Effects

We established three regression models to analyze the main effects of SP on WPH in the three datasets respectively (see Table 2). The results indicated that SP has significant and positive effects on WPH in Model 1 (*β* = 0.071, *p* < 0.01) and Model 2 (*β* = 0.147, *p* < 0.01) but a nonsignificant effect on WPH in Model 3 (*β* = −0.034, *p* = 0.512). These results suggest that the pandemic improved people’s WPH during the outbreak period of COVID-19. Namely, the more serious the pandemic was, the higher people’s WPH was. Moreover, such an effect was still at work in the short term after the pandemic had been under control for 6 months. However, this effect disappeared a year and a half later, which suggests that the promotion effect of the pandemic on people’s WPH can only be maintained in the short term and does not persist in the long term. Therefore, H1, H4a, H4b, and H4c have been verified. In addition, according to the results for Model 1, AMHI had a significant positive effect on WPH (*β* = 0.088, *p* < 0.05), which suggests that residents with a higher household income were more willing to pay for health during the outbreak period of the pandemic. Moreover, the education level had a marginally significant positive effect on WPH (*β* = 0.052, *p* = 0.059), indicating that residents with a higher education level might also have had a higher WPH during the outbreak period.

### 4.2. Analysis of Mediating Effects

The bootstrapping method was employed to test for these mediating effects using Model 4 of the PROCESS macro by Hayes for the three data sets separately (see Table 3) [34]. The bootstrap analysis of 5000 samples generated a 95% confidence interval (CI) for the indices of mediating effects. If the CI does not include 0, it indicates a significant mediating effect. Consistent with our hypotheses, the indirect effects of FD between SP and WPH were significant in the outbreak period data set (95% CI: 0.0149–0.0360) and the short-term data set (95% CI: 0.0035–0.0377) but nonsignificant in the long-term data set (95% CI: −0.0060–0.0268). These results suggest that the pandemic increased people’s FD and thus enhanced their WPH. Thus, H2 has been verified. However, such a mediating effect can only be verified in the outbreak period and the short-term period when the pandemic was under control.

### 4.3. Analysis of the Moderated Mediation Model

As the main effect and mediating effect were not significant in the long-term data set, we only analyzed the moderated mediation model, as set out in Figure 1, using the outbreak period and short-term post-pandemic data sets. The bootstrapping method was employed to test for the moderated mediations using Model 15 of the PROCESS macro by Hayes [34]. The interaction of FD and self-esteem had significant negative effects on WPH in the outbreak period data set (*β* = −0.113, *p* = 0.000) and short-term data set (*β* = −0.115, *p* = 0.014). These results indicate that self-esteem moderated the positive effect of FD on WPH. Specifically, the positive effect of FD on the subsequent WPH of people with low self-esteem was higher than that of people with high self-esteem. Thus, H3 has been verified. This moderating effect exists both during the outbreak period and in the short term after the pandemic.

## 5. Discussion

### 5.1. Theoretical Contributions

The theoretical contributions of the present study are mainly shown in the following aspects. First, using the method of follow-up research, our study demonstrated that the pandemic had a significant impact on people’s WPH, which persisted in the short term but subsided in the long term. Given that previous studies are primarily based on cross-sectional data from the outbreak period [13,14], the design of this longitudinal study is an important innovative contribution that can more clearly reveal how the COVID-19 pandemic affects people’s WPH over time. Second, based on TMT, we found that FD explained the effect of the pandemic on people’s WPH not only during the outbreak but also in the short period after the outbreak was controlled. This result indicates that the FD caused by the pandemic will continue for a notable period and will consequently affect people’s psychological state and behaviors. These findings may inform new theories for the development of the TMT. Finally, our study verified the moderating role of self-esteem. Specifically, when experiencing the FD caused by COVID-19, not all individuals will respond to the threat with healthy behaviors. People with high self-esteem, because they have more psychological resources, are less willing to pay for healthy ways to deal with the fear compared to people with low self-esteem. The proposed boundary condition of self-esteem not only complements and improves the mechanism of action studied herein but also provides a new theoretical understanding of the role of self-esteem.

Our conclusions can also inspire further research in the field of consumer behavior. The existing literature has found that consumers showed many irrational consumption behaviors in the early stage of the pandemic outbreak, for example, indulging in impulsive consumption [35,36], conformity consumption [37,38], and rushing to buy scarce products [39]. This irrational consumption primarily constitutes situational behavior and is caused by the consumers’ state of stress. With the pandemic becoming a long-term state, consumers’ coping strategies gradually change. Consumers become more committed to a rational response to the long-term threat of the pandemic, for example, by cutting spending [15,16], adopting sustainable consumption [40,41], and focusing on utilitarian purchases [42]. Such studies seem to imply that consumers’ rational coping strategies in response to the pandemic differ from their situational responses. However, the results of our study found that purchasing health goods, as a relatively rational consumption behavior, not only played an important role in buffering people’s fear in the outbreak period but also lasted for a certain period of time after the pandemic had been brought under control. Therefore, guiding consumers’ healthy consumption is beneficial for both the early and later stages of a pandemic.

### 5.2. Practical Implications

The results herein indicate that although COVID-19 has brought a great threat to people’s lives, it has also brought some positive effects. For example, the pandemic has promoted people’s WPH and encouraged them to pay more attention to their own health. However, the longitudinal study found that this effect only lasted in the short term; therefore, the health industry and relevant health promotion institutions should seize this opportunity to reinforce people’s health awareness, stimulate consumers’ enthusiasm for health-related product consumption, and promote the development of the health industry. By testing the moderating effect of self-esteem, we also found that compared to individuals with low self-esteem, those with high self-esteem were less inclined to consume such products to improve their health given the threat caused by the pandemic. This might be because for individuals with high self-esteem, taking defensive measures is a sign of weakness. In addition, it is worth noting that compared to Asian cultures, European and American cultures place more emphasis on independence, achievement, and self-esteem; thus, people in European and American countries have been less inclined to wear masks during the outbreak, which may also be an important cause of the rapid spread of COVID-19 in Europe and the United States. Our study argues that, particularly for individuals with high self-esteem, during a global public health crisis, promoting healthy behaviors should not be targeted from the point of view that consumers can passively compensate for their lack of health but from the point of view that the consumption of health-related products is the embodiment of consumers’ control over their health.

### 5.3. Limitations and Future Research Directions

Although the current study has the aforementioned theoretical contributions and practical value, it still has the following limitations. The data herein are mainly taken from China, and it is unclear whether the findings can be extrapolated globally. The pandemic is still ongoing in many countries at the time of writing, and values vary across different cultural contexts. In future studies, we aim to perform a comparison between different countries and regions.

## 6. Conclusions

Our study found that the COVID-19 pandemic promoted people’s WPH during the outbreak period. FD mediated this effect. Specifically, the pandemic increased people’s FD and, thus, enhanced their WPH. Self-esteem moderated the relationship between FD and WPH. In addition, the positive effect of FD on the WPH of people with low self-esteem was higher than that of the WPH of people with high self-esteem. This effect was sustained in the short term after the pandemic had been brought under control but not in the long term.

## Figures and Tables

**Table 1:** Reliability and validity analysis (*n* = 1548).

Variable Name	Item	Standardized Factor Loading	Cronbach’s α	CR	AVE
SP	CCC	0.615	0.885	0.864	0.619
	NCC	0.735			
	CSC	0.871			
	NSC	0.893			
WPH	W1	0.807	0.702	0.835	0.629
	W2	0.851			
	W3	0.716			
FD	F1	0.793	0.818	0.876	0.702
	F2	0.867			
	F3	0.852			
Self-esteem	SE1	0.651	0.878	0.919	0.532
	SE2	0.786			
	SE3	0.637			
	SE4	0.777			
	SE5	0.695			
	SE6	0.666			
	SE7	0.746			
	SE8	0.780			
	SE9	0.755			
	SE10	0.781			

Note: CR = composite reliability, AVE = average variance extracted, SP = severity of the pandemic, WPH = willingness to pay for health, FD = fear of death, CCC = the cumulative number of confirmed cases per city, NCC = the number of new confirmed cases per city, CSC = the cumulative number of suspected cases per city, NSC = the number of new suspected cases per city, W1–W3 = the first item to the third item of WPH, F1–F3 = the first item to the third item of FD, SE1–SE10 = The first item to the tenth item of Self-esteem.

**Table 2:** Main effects of SP on WPH.

Data Set	Outbreak Period (*n* = 1548)		Short Term (*n* = 466)		Long Term (*n* = 427)	
Model	Model 1		Model 2		Model 3	
Dependent Variable	WPH		WPH		WPH	
Statistics	*β*	*p*	*β*	*p*	*β*	*p*
**Control Variable**						
*Gender (female* *=* *0)*	−0.014	0.612	0.058	0.250	−0.028	0.604
*Age*	0.006	0.836	0.043	0.402	0.005	0.935
*AMPI*	0.029	0.445	0.024	0.713	0.061	0.433
*AMHI*	0.088 *	0.013	0.015	0.811	0.128	0.058
*AMPE*	0.014	0.719	0.054	0.408	0.037	0.614
*AMHE*	0.057	0.164	0.024	0.730	−0.076	0.338
*Education*	0.052	0.059	0.036	0.480	0.079	0.136
**Independent Variable**						
*SP*	0.071 **	0.009	0.147 **	0.003	−0.034	0.512
**Model Summary**						
*R^2^*	0.042		0.047		0.032	
*Adj.R^2^*	0.036		0.028		0.012	
*F*	7.728 ***		2.579 **		1.578	

Note: * *p* < 0.05, ** *p* < 0.01, *** *p* < 0.001; WPH = willingness to pay for health, SP = severity of the pandemic, AMPI = average monthly personal income, AMHI = average monthly household income, AMPE = average monthly personal expenses, AMHE = average monthly household expenditure.

**Table 3:** Mediating effects of FD between SP and WPH.

Data Set	Name of Effects	Effect	SE	LLCI	ULCI
Outbreak Period (*n* = 1548)	Total Effect	0.0568	0.0216	0.0145	0.0991
	Direct Effect	0.0325	0.0214	−0.0095	0.0746
	Indirect Effect	0.0242	0.0054	0.0149	0.0360
Short Term (*n* = 466)	Total Effect	0.1041	0.0352	0.0348	0.1734
	Direct Effect	0.0885	0.0354	0.0188	0.1581
	Indirect Effect	0.0156	0.0084	0.0035	0.0377
Long Term (*n* = 427)	Total Effect	−0.0073	0.0388	−0.0837	0.0690
	Direct Effect	−0.0145	0.0382	−0.0896	0.0607
	Indirect Effect	0.0071	0.0081	−0.0060	0.0268

Note: SE = standard error, LLCI = lower level of 95% confidence interval, ULCI = upper level of 95% confidence interval.

## Data Availability

The data can be obtained by contacting the corresponding author.

## Appendix A

**Table A1:** Items of Rosenberg’s Self-Esteem Scale.

1. On the whole, I am satisfied with myself.
2. At times, I think that I am no good at all.
3. I feel that I have a number of good qualities.
4. I am able to do things as well as most other people.
5. I feel that I do not have much to be proud of.
6. I certainly feel useless at times.
7. I feel that I am a person of worth, at least on an equal level with others.
8. I wish that I could have more respect for myself.
9. All in all, I am inclined to feel that I am a failure.
10. I take a positive attitude toward myself.
